# Razão Troponina/Linfócitos: Antecipando a Miocardite Causada por Imunoterapia

**DOI:** 10.36660/abc.20250867

**Published:** 2026-04-14

**Authors:** Inês Miranda, João Bicho Augusto

**Affiliations:** 1 Hospital Prof. Dr. Fernando Fonseca Serviço de Cardiologia Amadora Portugal Serviço de Cardiologia, Hospital Prof. Dr. Fernando Fonseca, Amadora – Portugal; 2 Faculdade de Medicina da Universidade de Lisboa Lisboa Portugal Faculdade de Medicina da Universidade de Lisboa, Lisboa – Portugal; 3 Hospital CUF Tejo Centro de Coração e Vasos Lisboa Portugal Centro de Coração e Vasos, Hospital CUF Tejo, Lisboa – Portugal; 4 Universidade Católica Portuguesa Católica Medical School Lisboa Portugal Católica Medical School, Universidade Católica Portuguesa, Lisboa – Portugal; 5 University College London Institute of Cardiovascular Science Londres Inglaterra Institute of Cardiovascular Science, University College London, Londres – Inglaterra

**Keywords:** Inibidores de Checkpoint Imunológico, Miocardite, Relação Troponina/linfócito, Biomarcadores, Cardio-oncologia

Os inibidores de checkpoint imunológico (ICI) são amplamente utilizados no tratamento de cânceres agressivos, como câncer de pulmão e melanoma, e revolucionaram a terapia oncológica, oferecendo respostas duradouras em neoplasias agressivas. No entanto, como qualquer outra terapia oncológica, existe a preocupação com os potenciais eventos adversos cardíacos. No caso dos ICI, a miocardite é um evento adverso imunomediado (EAI) conhecido, porém raro, grave e potencialmente fatal, com uma taxa de mortalidade de até 50% em casos graves.^1^ Houve algumas tentativas anteriores de identificar pacientes com alto risco de desenvolver miocardite associada a ICI, integrando marcadores clínicos e laboratoriais, como elevação de troponina,^1,2^ níveis de creatina quinase e NT-proBNP^3^ e alterações no eletrocardiograma,^4^ que já são familiares na avaliação de pacientes com miocardite em um contexto clínico não relacionado à terapia oncológica. Mais recentemente, modelos de risco compostos baseados em aprendizado de máquina têm sido explorados. Por exemplo, um algoritmo desenvolvido por Awadalla et al.^5^ integrou alterações de troponina, variações no eletrocardiograma e imagens de deformação ecocardiográfica para prever o risco de miocardite, superando as abordagens tradicionais baseadas em um único biomarcador. Apesar desses avanços, nenhuma ferramenta de estratificação de risco validada prospectivamente é atualmente utilizada rotineiramente na prática clínica para prever pacientes de alto risco.

No entanto, é necessário estratificar esses pacientes de forma rápida e eficiente para compreender melhor quem precisa de maior atenção e orientar a triagem e o monitoramento pré-tratamento. O potencial de utilização de biomarcadores prognósticos permanece promissor, por se tratar de uma forma rápida e custo-eficiente de avaliar pacientes. Neste número dos Arquivos Brasileiros de Cardiologia, Chen et al. abordaram esse problema utilizando a razão troponina cardíaca/contagem absoluta de linfócitos (cTn/ALC) para identificar pacientes de alto risco mais precocemente no processo da doença.^6^ Os autores compararam retrospectivamente pacientes com miocardite associada à ICI a controles sem complicações cardiovasculares relacionadas à ICI, concluindo que os pacientes que desenvolveram miocardite apresentavam uma relação cTn/ALC mais elevada, prevendo desfechos desfavoráveis nesse cenário. Isso está em consonância com estudos anteriores que sugerem que biomarcadores são essenciais na estratificação e no acompanhamento de pacientes com câncer submetidos a terapias com ICI,^7,8^ com cTn elevada associada a piores desfechos na miocardite induzida por ICI; além disso, a linfopenia é um marcador de desregulação imunológica, frequentemente observada em pacientes com câncer submetidos à terapia com ICI^4^ e associada a desfechos desfavoráveis em uma variedade de EAIs.^9^

Dada a complexa fisiopatologia da miocardite induzida por ICI, que se acredita ser impulsionada por uma inflamação aberrante mediada por células T que atinge o tecido miocárdico,^10^ é intuitivo que busquemos utilizar biomarcadores familiares, de baixo custo e que representem danos imunológicos e aos cardiomiócitos, combinados ou separadamente. Outras estratégias também podem auxiliar no diagnóstico e na avaliação desses pacientes, como a ressonância magnética cardíaca e/ou a biópsia endomiocárdica, mas, embora úteis, nem sempre estão disponíveis. Isso torna ainda mais importante encontrar biomarcadores acessíveis e rápidos que possam auxiliar na detecção precoce e na triagem. A relação cTn/ALC se apresenta como uma candidata promissora, estando disponível em exames de sangue comuns e já fazendo parte da investigação padrão para pacientes com câncer que apresentam sintomas cardíacos potenciais.

Apesar de parecerem simples e promissores, o uso generalizado desses biomarcadores não está isento de preocupações. Muitos dos estudos são unicêntricos e retrospectivos, com amostras relativamente pequenas, o que pode limitar a generalização dos resultados. A validação prospectiva em coortes multicêntricas maiores é necessária antes que o uso em larga escala de biomarcadores como a razão cTn/ALC possa ser adotado na prática clínica de rotina. É importante lembrar que esses pacientes geralmente são complexos, com diversas diferenças clínicas e heterogeneidade, e que os níveis dos biomarcadores podem flutuar e apresentar variações não apenas relacionadas à imunoterapia com ICI. Há também uma variabilidade substancial na sensibilidade e especificidade desses marcadores para miocardite induzida por ICI. Por exemplo, embora a troponina T de alta sensibilidade seja amplamente utilizada, ela pode estar elevada em diversas condições cardíacas e sistêmicas, reduzindo sua especificidade em pacientes com câncer e patologias sobrepostas, e os limiares ideais nesse contexto permanecem indefinidos.^1,2,11^ Medições seriadas também podem oferecer informações adicionais sobre a progressão da doença ou a resposta à terapia.^12^

Outra limitação é a falta de protocolos padronizados para a medição seriada de biomarcadores e sua integração com a tomada de decisões clínicas. Embora algumas instituições defendam o monitoramento de rotina em pacientes de alto risco, essa abordagem não é universalmente adotada e faltam evidências de custo-efetividade ou de melhoria dos desfechos. Além disso, a relação temporal entre a elevação dos biomarcadores e o início ou a gravidade da miocardite é pouco compreendida, o que dificulta os esforços para utilizá-los no diagnóstico precoce ou na definição do prognóstico.

Olhando para o futuro, essas descobertas levantam questões interessantes para pesquisas futuras. Biomarcadores como a razão cTn/ALC poderiam ajudar a identificar miocardite subclínica antes do desenvolvimento de sintomas evidentes? Poderiam auxiliar na orientação do início ou da redução gradual da terapia com corticosteroides? Mais ainda, poderiam eventualmente desempenhar um papel importante ao ajudar os médicos a ponderar os riscos e benefícios da retomada da imunoterapia com ICI após a resolução da miocardite, ou mesmo prever recidivas ou sequelas a longo prazo? À medida que a ICI se expande para estágios iniciais do câncer e é combinada com mais frequência a outras terapias, se espera que a incidência de eventos adversos imunomediados (incluindo miocardite) aumente.^5^ Há uma necessidade urgente de ferramentas eficientes de estratificação de risco. Painéis de biomarcadores, juntamente com avaliação clínica, exames de imagem e outros dados laboratoriais, provavelmente são o caminho a seguir, mas escores compostos que integrem todas essas variáveis podem ser úteis para a tomada de decisões clínicas. Preencher essas lacunas é essencial para refinar a estratificação e o manejo de risco para pacientes que recebem terapia com ICI.
