# Supplementary material for: Razão Troponina/Linfócitos: Antecipando a Miocardite Causada por Imunoterapia
Source: Arq Bras Cardiol. 2026 Apr 14;122(8):e20250867. [Article in Portuguese] doi: 10.36660/abc.20250867 (PMC13155792; doi:10.36660/abc.20250867)
Supplement: Translation

# Translation: Troponin/Lymphocyte Ratio: Anticipating Immunotherapy-Induced Myocarditis

**DOI:** 10.36660/abc.20250867i

**Authors:** Inês Miranda, João Bicho Augusto

**Affiliations:** 1 Amadora Portugal Serviço de Cardiologia, Hospital Prof. Dr. Fernando Fonseca, Amadora – Portugal; 2 Lisboa Portugal Faculdade de Medicina da Universidade de Lisboa, Lisboa – Portugal; 3 Lisboa Portugal Centro de Coração e Vasos, Hospital CUF Tejo, Lisboa – Portugal; 4 Lisboa Portugal Católica Medical School, Universidade Católica Portuguesa, Lisboa – Portugal; 5 London England Institute of Cardiovascular Science, University College London, London – England

**Keywords:** Immune Checkpoint Inhibitors, Myocarditis, Troponin-to-lymphocyte Ratio, Biomarkers, Cardio-oncology

Immune checkpoint inhibitors (ICI) are widely used in treating aggressive cancers like lung cancer and melanoma, and they have revolutionized cancer therapy, offering durable responses in aggressive malignancies. However, like any other cancer therapy, there is a concern with potential cardiac adverse outcomes. In the case of ICI, myocarditis is a known, yet rare, severe, and potentially fatal immune-related adverse event (irAE) with a mortality rate of up to 50% in severe cases.^1^ There have been some previous attempts to identify patients at high risk of developing ICI-associated myocarditis by integrating clinical and laboratory markers like troponin elevation,^1,2^ creatine kinase and NTproBNP levels,^3^ and electrocardiogram changes,^4^ which are already familiar to us when evaluating myocarditis patients in a clinical scenario not related to cancer therapy. More recently, machine-learning composite risk models have been explored. For instance, an algorithm developed by Awadalla et al.^5^ integrated troponin changes, electrocardiogram variations, and echocardiographic strain imaging to predict myocarditis risk, outperforming traditional single-biomarker approaches. Despite these advances, no prospectively validated risk stratification tool is currently routinely used in clinical practice to predict high-risk patients.

There is, however, a need to quickly and efficiently stratify these patients to better understand who requires higher vigilance and to guide pre-treatment screening and monitoring. The potential use of prognostic biomarkers remains promising, being a quick and cost-efficient way to evaluate patients. In this issue of *Arquivos Brasileiros de Cardiologia*, Chen et al. tackled this problem by using the cardiac troponin-to-absolute lymphocyte count (cTn/ALC) ratio to identify high-risk patients earlier in the disease process.^6^ The authors retrospectively compared ICI-associated myocarditis patients to controls without ICI-related cardiovascular complications, concluding that patients who developed myocarditis had a higher cTN/ALC ratio, predicting poor outcomes in this scenario. This is in line with previous studies that suggest biomarkers are essential in the stratification and follow-up of cancer patients receiving ICI-therapies,^7,8^ with elevated cTn associated with worse outcomes in ICI myocarditis; furthermore, lymphopenia is a surrogate for immune dysregulation, often seen in cancer patients undergoing ICI therapy^4^ and associated with poor outcomes in a variety of irAE.^9^

Given the complex pathophysiology of ICI myocarditis, which is believed to be driven by aberrant T-cell–mediated inflammation that targets myocardial tissue^10^ it is intuitive that we try to use biomarkers that are familiar, low-cost, and that represent immune and cardiomyocyte damage, either combined or apart. Other strategies can also help in the diagnosis and evaluation of these patients, like cardiac MRI and/or endomyocardial biopsy, but, while useful, they are not always available. This makes it even more important to find accessible and rapid biomarkers that can aid in early detection and triage. cTn/ALC ratio presents a promising candidate, being available on commonly obtained blood tests and already a part of standard workups for cancer patients in those presenting with potential cardiac symptoms.

Despite looking simple and promising, widespread use of these biomarkers does not come without concern. Many of the studies are single-center and retrospective with a relatively small sample size, which may limit the generalization of these findings. Prospective validation in larger, multicenter cohorts is required before large-scale use of biomarkers like cTn/ALC ratio can be adopted into routine clinical practice. It is important to remind ourselves that these are usually complex patients with several clinical differences and heterogeneity, and that biomarker levels may fluctuate and present variations not only related to ICI. There is also substantial variability in the sensitivity and specificity of these markers for ICI myocarditis. For instance, while high-sensitivity troponin T is widely used, it may be elevated in various cardiac and systemic conditions, reducing its specificity in cancer patients with overlapping pathologies, and optimal thresholds in this context remain undefined.^1,2,11^ Serial measurements may also offer additional insight into disease progression or response to therapy.^12^

Another limitation is the lack of standardized protocols for serial biomarker measurement and integration with clinical decision-making. While some institutions advocate routine monitoring in high-risk patients, this approach is not universally adopted, and evidence for cost-effectiveness or improved outcomes is lacking. Additionally, the temporal relationship between biomarker elevation and the onset or severity of myocarditis is poorly understood, which complicates efforts to use them for early diagnosis or prognostication.

Looking ahead, these findings raise interesting questions for future research. Could biomarkers like the cTn/ALC ratio help identify subclinical myocarditis before overt symptoms develop? Could they help in guiding steroid initiation or tapering strategies? Even more, could they eventually play a role in helping clinicians weigh the risks and benefits of restarting ICI after myocarditis resolution or even predict relapses or long-term sequelae? As ICI expands into earlier-stage cancers and becomes more frequently combined with other therapies, the incidence of irAE (including myocarditis) is expected to rise.^5^ There is an urgent need for efficient risk stratification tools. Biomarker panels alongside clinical judgment, imaging, and other laboratory data are probably the way to go, but composite scores that integrate all these variables might be useful for clinical decision-making. Addressing these gaps is essential to refining risk stratification and management for patients receiving ICI therapy.
